# Hypoxia-induced metabolic reprogramming in mesenchymal stem cells: unlocking the regenerative potential of secreted factors

**DOI:** 10.3389/fcell.2025.1609082

**Published:** 2025-06-09

**Authors:** Wendy V. Jaraba-Álvarez, Ashanti C. Uscanga-Palomeque, Vanesa Sanchez-Giraldo, Claudia Madrid, Hector Ortega-Arellano, Karolynn Halpert, Carolina Quintero-Gil

**Affiliations:** ^1^ BioXscience, BioXcellerator, Medellín, Colombia; ^2^ BioXtech, Medellín, Colombia

**Keywords:** mesenchymal stem cells (MSC), regenerative medicine, hypoxia preconditioning, cellular microenvironment, extracellular vesicles (EV), mitochondria

## Abstract

Mesenchymal stem cells (MSCs) are a cornerstone of regenerative medicine, primarily due to their ability to secrete bioactive factors that modulate inflammation, promote tissue repair, and support regeneration. Recent research highlights the importance of preserving the native cellular microenvironment to optimize MSC function and survival post-transplantation. Preconditioning strategies, such as hypoxia exposure, have emerged as powerful tools to enhance MSC therapeutic potential by mimicking physiological conditions in their natural niche. This perspective article explores the metabolic adaptations induced by hypoxia in MSCs, focusing on shifts in mitochondrial function, glycolysis, oxidative phosphorylation, and metabolic intermediates that enhance cellular survival and bioactivity. We also discuss how these metabolic changes influence the composition and function of MSC-derived secreted factors, particularly exosomes and other extracellular vesicles, in modulating tissue repair. Furthermore, we provide an overview of preclinical and clinical studies that have evaluated hypoxia-preconditioned MSCs and their byproducts, assessing their efficacy in various therapeutic contexts. Special attention is given to the role of hypoxia-induced mitochondrial adaptations in improving MSC function and the emerging potential of metabolic inhibitors or respiration modulators as strategies to further refine MSC-based therapies. By integrating metabolic insights with clinical evidence, we aim to offer a comprehensive perspective on optimizing MSC culture conditions to enhance their regenerative properties, acknowledging that this remains a theoretical standpoint, as conventional culture methods are generally not conducted under hypoxic conditions. This approach holds promise for the development of more effective therapeutic strategies that leverage metabolic modulation to improve MSC-based interventions for a range of diseases.

## 1 Introduction

Regenerative medicine has emerged as a therapeutic option to repair or replace damaged tissues or organs, using biomaterials, biomolecules, and cells, particularly adult stem cells (Armstrong et al., 2020). In the late 90s, a paradigm shift was necessary to understand that not only hematopoietic cells were adult stem cells, but that many other sources of adult stem cells can function by supporting the repair and regeneration of differentiated cells that naturally expire or are injured, rather than directly replacing them. These cells known as mesenchymal stem cells (MSCs), mesenchymal stromal cells or medicinal signaling cells (Caplan, 2010; Samsonraj et al., 2017) play a vital role in the field of regenerative medicine because of their multipotent nature, which allows them to differentiate into the mesodermal lineage and create new tissue such as bone, muscle, and skin (Samsonraj et al., 2017). Other primary functions of MSCs are immunomodulation, autocrine and paracrine activities, and evasion of innate immunity (Armstrong et al., 2020). These functions depend on the level of stimulation the cell receives, which has made the effects of the cellular microenvironment not only on MSCs efficacy but also on their ability to secrete factors, an attractive research field (Lotfy, AboQuella, and Wang, 2023).

In regenerative medicine, particularly cell-based therapies, maintaining the cellular microenvironment during cell culturing is one of the major challenges, because consistency in the composition and viability is needed (Fears et al., 2021), particularly for large scale production of cell-based products (Terzic et al., 2015). The microenvironment plays a crucial role in regulating stem cell behavior, including their proliferation, differentiation, and survival. The conditions under which stem cells are cultured, such as nutrient supply, oxygen levels, and substrate stiffness, directly influence their therapeutic potential, and ideally, the physiological niche should be considered, and efforts should be made to simulate these conditions *in vitro*, to enhance the adaptation and functionality of MSCs when administered, as well as to prevent risks related to the infusion of poorly characterized cells (Galipeau et al., 2021). In some cell therapy models, the evaluation of specific surface marker expression is essential, such as CD142, which has been associated with a pro-thrombotic effect, therefore, it must be considered whether the therapeutic use of MSCs will involve venous infusion (Moll et al., 2022), another example is hypoxic preconditioning and the selection of an appropriate extracellular matrix, both of which have been shown to make cell therapy more effective and safer in *in vivo* preclinical models (Yang et al., 2022). Despite this evidence, traditional cell culture methods have typically been optimized for laboratory conditions, but they often do not faithfully reproduce the physiological microenvironment in which cells will perform their *in vivo* function (Mas-Bargues et al., 2019). Therefore, a shift in focus from culture methods that mimic tissue function could allow the production of cells that can target affected sites and induce their therapeutic effect more effectively.

One of the most important considerations of the physiological environment is oxygen concentration, which varies according to each niche and cellular function, from 1% in the human eye to 14% in the lungs. However, traditional culture methods do not use devices to control oxygen concentrations and work is done under normal ambient oxygen conditions of 21% (normoxia), which represents a hyperoxia condition considering the hypoxic origin of most cell lines (Mas-Bargues et al., 2019), which can have consequences on various functions and stages of the cell cycle and affect growth, multiplication, differentiation and gene expression profile (Liu et al., 2017). In recent years, different studies have pointed out the importance of cell culture under optimal oxygen conditions for MSCs. However, there is still no consensus, so below, we will provide some examples of the advantages at the cellular level of hypoxic cultures and how these may contribute to greater therapeutic success in the field of regenerative medicine. Specifically, we will explore how enhancing therapeutic potential through hypoxia exposure can optimize MSC function. We will also discuss preclinical and clinical studies on the efficacy of hypoxia-preconditioned MSCs, highlighting their improved regenerative properties. Additionally, we will examine the role of hypoxia-derived exosomes and extracellular vesicles in facilitating tissue repair, and the impact of hypoxia on mitochondrial modulation in MSCs, which plays a crucial role in their metabolic adaptation and therapeutic effectiveness.

## 2 Enhancing therapeutic potential through hypoxia exposure

**FIGURE 1:**
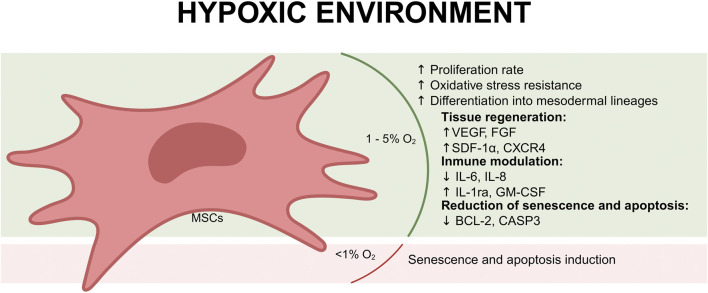
Effects of a hypoxic environment (1%–5% O_2_) on mesenchymal stem cells (MSCs). Under mild hypoxia, MSCs exhibit increased proliferation, enhanced oxidative stress resistance, and improved differentiation into mesodermal lineages. Hypoxia promotes tissue regeneration by upregulating key growth factors (VEGF, FGF, SDF-1α, and CXCR4), which support angiogenesis and repair processes. Additionally, MSCs under hypoxic conditions display immunomodulatory effects, suppressing pro-inflammatory cytokines (IL-6, IL-8) while increasing anti-inflammatory factors (IL-1ra, GM-CSF). Hypoxia also reduces senescence and apoptosis by downregulating pro-apoptotic genes (BCL-2, CASP3). However, exposure to extreme hypoxia (<1% O_2_) can induce senescence and apoptosis, compromising MSC viability and therapeutic potential.

Culturing mesenchymal stem cells (MSCs) in hypoxic conditions has emerged as a promising strategy to enhance their therapeutic potential because hypoxia alters MSCs transcriptional profile, promotes their proliferation, and increases the production of EVs (Yuan et al., 2025). MSCs, are naturally found in tissues under low oxygen tension, such as bone marrow, adipose tissue, and other connective tissues (Keith and Simon, n. d.). The physiological oxygen levels within these niches are typically around 6%–7% O2, which is significantly lower than atmospheric oxygen levels (21%) (Mas-Bargues et al., 2019). Recent studies reported that MSCs cultured in a hypoxic environment may enhance the immunomodulatory capacity of MSCs and elevate the expression of angiogenic factors, pro-survival proteins, superior vascularization effects, and anti-apoptotic/anti-aging proteins for enhanced cellular protection and regeneration (Figure 1). On the other hand, severe hypoxia (<1% O2) negatively affects the *in vitro* therapeutic potential of MSCs and causes their senescence and apoptosis (Khasawneh and Abu-El-Rub, 2022).

On the molecular level, the hypoxia-inducible factor 1-alpha (HIF-1α) plays a pivotal role in the cellular response to low oxygen conditions. Under hypoxic conditions, HIF-1α is stabilized and translocated to the nucleus, where it activates the transcription of genes involved in cellular adaptation to oxygen deprivation, including those responsible for angiogenesis, cell survival, and metabolism (Yusoff et al., 2022). In MSCs, HIF-1α activation has been shown to enhance the colony-forming potential, self-renewal, and differentiation capabilities of these cells. Moreover, HIF-1α promotes the upregulation of VEGF and SDF-1α, which are essential for both MSC homing to injury sites and the initiation of tissue repair processes (Yusoff et al., 2022).

Another important aspect of hypoxia-preconditioned MSCs is their enhanced ability to home to injury sites. MSCs exposed to hypoxic conditions show increased expression of chemokine receptors, particularly CXCR4, which plays a crucial role in MSC migration toward areas of tissue damage. This enhanced homing ability is attributed to the upregulation of SDF-1α, a chemokine that is released at injury sites and guides MSCs to the damaged tissues (Dai et al., 2017). This process is essential for the effective delivery of MSCs to areas of tissue injury, where they can exert their reparative effects. It is also crucial to control the exposure time of MSCs to hypoxic conditions. Studies suggest that the optimal exposure time is less than 48 h, which favors the activation of protective mechanisms without causing significant cellular damage. Longer exposures can trigger accelerated cellular aging, thus reducing the therapeutic efficacy of MSCs (Kwon et al., 2017).

## 3 Preclinical and clinical studies on the efficacy of hypoxia preconditioned MSCs

In various preclinical models, hypoxia-preconditioned MSCs have demonstrated superior outcomes compared to their normoxic counterparts. For example, in a rat model of massive hepatectomy, hypoxia-preconditioned bone marrow MSCs enhanced liver regeneration, possibly by upregulating VEGF levels (Wang et al., 2024). Similarly, in a nonhuman primate model of myocardial infarction, hypoxia-preconditioned MSCs led to significant improvements in cardiac function and reduced infarct size without increasing arrhythmogenic risks, likely due to enhanced paracrine activity (Song et al., 2022).

While these preclinical findings are promising, clinical data on the efficacy of hypoxia-preconditioned WJ-MSCs remain limited (Le et al., 2024). After searching clinicaltrials.org, one study using Hypoxic Adipose tissue MSCs for the treatment of posterior cruciate ligament injury (NCT04889963) was found, but no results or updates are available. Also, two clinical trials using conditioned media from hypoxic cultured MSCs for treating knee osteoarthritis (NCT06688318) and severe COVID-19 (NCT04753476) were also found, both without results.

In a study from China, researchers explored hypoxia-preconditioned olfactory mucosa mesenchymal stem cells (hOM-MSC) to improve recovery in Parkinson’s disease (PD). They discovered that TGF-β1 secreted by these cells enhances mitochondrial function in dopaminergic neurons by modulating microglial immune responses and autophagy in the substantia nigra. Using techniques like scRNA-seq and ATAC-seq, they demonstrated that hOM-MSC shifts microglia from a pro-inflammatory (M1) to an anti-inflammatory (M2) state via the PI3K-Akt pathway. In a PD mouse model, hOM-MSC facilitated functional recovery and reduced neuronal oxidative stress. In a clinical trial involving five PD patients, hOM-MSC transplantation also resulted in reduced medication and improved motor function, as assessed by clinical scales and biomarkers (Zhuo et al., 2024).

More clinical studies are needed to assess the safety and efficacy of hypoxia-preconditioned MSCs in regenerative medicine.

## 4 Hypoxia-derived exosomes and extracellular vesicles

In addition to these cellular and molecular responses, hypoxia-induced MSCs have been shown to secrete higher levels of extracellular vesicles (EVs), which are membrane-bound particles that range in size from 30 to 150 nm, carry a variety of bioactive molecules, including proteins, lipids, and nucleic acids, which can modulate the behavior of neighboring and distant cells. The ability of exosomes to transfer functional genetic material, such as mRNA and non-coding RNAs, allows them to influence both intracellular processes, such as metabolism and signaling, as well as intercellular interactions, promoting tissue repair and regeneration (Raposo and Stoorvogel, 2013). Recent studies have highlighted the role of EVs in facilitating tissue regeneration in various injury models, such as renal ischemia-reperfusion injury (Yuan et al., 2025) and acute severe pancreatitis (Hu et al., 2023). The therapeutic potential of hypoxia-preconditioned MSCs, therefore, extends beyond their direct differentiation and tissue repair capabilities, as their secreted factors can modulate the local and distant microenvironment and facilitate healing processes.

The synergistic effects of combining hypoxia-preconditioned MSCs and their exosomes in therapeutic applications are significant. Studies have shown that the combined use of MSCs and exosomes derived from hypoxic conditions can produce more effective outcomes in terms of tissue repair, immune modulation, and cell proliferation. This approach takes advantage of the enhanced bioactive cargo carried by the exosomes, which not only supports MSC proliferation and survival but also helps to manage oxidative stress and promote tissue regeneration (Williams et al., 2023). Moreover, hypoxia-induced changes in exosomal content can improve cell homing and migration, which are essential for effective tissue repair and regeneration.

Wharton’s jelly mesenchymal stem cells (WJ-MSCs) from umbilical cord tissue are one of the most attractive sources for use in regenerative medicine, due to their ease of isolation and *in vitro* proliferation, the latter being favored by the hypoxic environment. For this reason, the effects of MSC cultivation under hypoxic conditions in various disease models have started to be studied, and one of the most promising results has been observed in central nervous system diseases, particularly due to the paracrine activity of WJ-MSCs and the release of neuroregulatory factors (Teixeira et al., 2015). In this study, Teixeira et al. demonstrated that the hypoxic environment did not affect cell viability, and that both under normoxia and hypoxia, WJ-MSCs were able to differentiate into “human CNS-derived cells”. Similarly, our research group demonstrated the transdifferentiation of WJ-MSCs into the ectodermal lineage by cultivating them in cerebrospinal fluid and measuring neuronal differentiation markers such as MAP2, NFL, and NeuN (Sánchez-Giraldo et al., 2023).

Additionally, Teixeira et al. demonstrated changes in the secretome of cells cultured under hypoxic conditions, with one of the most significant being the increased secretion profile of neuroregulatory molecules in WJ-MSCs cultured under hypoxia compared to those cultured under normoxia (Teixeira et al., 2015). Other recent studies also highlight the potential of EVs derived from various sources of mesenchymal stem cells (MSCs) to enhance angiogenesis, which could be of great importance in tissue regeneration and the treatment of cardiovascular diseases (Pulido-Escribano et al., 2022). However, although studies evaluating different exposure times and oxygen concentrations exist, standardization and consensus are necessary, as any variation in culturing conditions may influence the content and therapeutic properties of EVs.

Our research has demonstrated that hypoxic conditions significantly increase the production of exosomes by MSCs, particularly in the context of Wharton’s jelly-derived MSCs (WJ-MSCs). Under normoxic conditions, WJ-MSCs release approximately one–1.5 billion EVs per liter of conditioned media, while hypoxic cells produce a much larger quantity, ranging from 10 to 17 bil

Lion EVs per liter. This increase in exosome production under hypoxia is accompanied by an alteration in the size distribution of the EVs (Franco et al., 2025). In normoxic conditions, around 70% of the EVs fall within the size range of exosomes (100–300 nm), whereas hypoxic conditions result in 85% of the EVs meeting the exosomal size criteria. These findings suggest that hypoxia not only increases the quantity of exosomes but also may influence their characteristics, potentially enhancing their therapeutic efficacy (Williams et al., 2023).

Proteomic analysis of exosomes derived from hypoxic WJ-MSCs revealed differential expression of proteins associated with various biological processes, including inflammation, cell growth, collagen organization, and neurogenesis. Specifically, we identified 17 proteins that were significantly upregulated or downregulated in exosomes from hypoxic conditions compared to those derived from normoxic cells. These proteins are involved in key processes such as actin fiber organization, which is crucial for cell motility and tissue remodeling, and the regulation of neurogenesis, which is important for repairing nervous tissue (Franco et al., 2025). The presence of these proteins in exosomes highlights their potential for enhancing the regenerative capacity of MSC-based therapies, particularly in the context of tissue injuries where inflammation and cell migration are pivotal (Mu et al., 2022).

## 5 Hypoxia and mitochondrial modulation in MSCs

Mitochondria are often called the powerhouses of the cell because they generate the majority of the cell’s energy through cellular respiration, playing a crucial role in preserving overall cell health (Glancy et al., 2020). Dysfunction in mitochondria has been linked to cellular aging and several human diseases (Mohammadalipour, Dumbali, and Wenzel, 2020) like Mitochondrial Encephalopathy, Lactic Acidosis, and Stroke-like Episodes (MELAS) Syndrome, Leigth Syndrome and other such diabetes, Parkinson’s and Alzheimer’ diseases. Mitochondria also regulate a range of cellular functions, including apoptosis, autophagy, cell cycle regulation, differentiation, and aging (Rodriguez et al., 2018) becoming the mitochondria and key organelle for cellular adaptation to physiological and pathological microenvironments (Mohammadalipour, Dumbali, and Wenzel, 2020).

The transfer of mitochondria between cells, a mechanism through which damaged or dysfunctional cells signal mesenchymal stem cells to supply healthy mitochondria, has been found to restore mitochondrial function in tissues such as neurons, cardiomyocytes, renal tubular epithelial cells and corneal epithelium (Thomas et al., 2022). The intercellular mitochondrial transport occurs through several mechanisms, including tunneling nanotubes (TNTs), gap junction channels, cell adhesion-mediated, cell fusion and EVs (Mukkala et al., 2023, Malekpour et al., 2023).

As mentioned before, hypoxia alters MSCs transcriptional profile, promotes their proliferation, and increases the production of EVs (Yuan et al., 2025). Since hypoxia increases EVs and EVs contain mitochondria we hypothesize that preconditioning of MSCs could release more mitochondria that can be used by damaged cells in tissues to be repair. Indeed, a study of acute severe pancreatitis (SAP), hypoxic preconditioning MSCs (5% O2, Hypo-MSCs) showed that the EVs had mitochondria. The therapeutic effect of EVs was significantly diminished after the inhibition of mitochondrial function with rhodamine 6G. This suggests a critical role for mitochondrial function in MSC-EVs for the treatment of SAP (Hu et al., 2023).

An emerging benefit of mesenchymal stem cells (MSCs) is their ability to enhance mitochondrial function in injured tissues by promoting efficient mitochondrial quality control (MQC) (Mukkala et al., 2023). Further research is required to elucidate whether modifications in the preconditioning culture of MSCs, such as hypoxia, can effectively enhance mitochondrial quality.

## 6 Discussion

Culture conditions and the cellular microenvironment can help enhance the effects of cell therapy. However, simulating and adequately maintaining physiological characteristics remains a significant challenge in the laboratory, as molecular-level changes can occur that favor signaling pathways that enhance cellular attributes and their regenerative potential, or conversely, stress signals and cell death may be triggered (Khasawneh and Abu-El-Rub, 2022).

In recent years, the hypoxic microenvironment (1%–5% O_2_) has been investigated for the cultivation of MSCs, with favorable outcomes observed in terms of immunomodulatory activity, cell viability, and proliferation. Nevertheless, these observations remain primarily limited to *in vitro* studies, and hypoxia has not yet been adopted as a routine culture condition in the preparation of advanced therapy medicinal products. However, hypoxic conditions also promote changes in metabolic activity by inducing the Cori cycle, in which pyruvate and lactate are metabolized to generate two molecules of ATP and mitochondrial reactive oxygen species (mtROS). These mtROS can have a beneficial effect by helping to combat stress during adaptation to the hypoxic environment and promoting signaling cascades via PI3K/Akt/mTOR, which increase cell proliferation (Liu et al., 2017).

Regarding culture conditions, traditional 2D surfaces have been the preferred choice due to the adherent phenotype of MSCs. However, there has been a recent shift towards the use of 3D culture methods, in which cells can grow in agitation, adhering to microspheres made of various materials, which may or may not be biodegradable, or as spheroids (Toghiani et al., 2024). These 3D methods offer two main advantages: one related to the production and large-scale expansion required for therapeutic use, and another related to the ease of collecting conditioned media, thus facilitating the purification of extracellular vesicles (EVs). Toghiani et al. reported a 3D culture model using spheroids under hypoxic conditions, finding that these conditions improved EV production, particularly those containing active molecules such as miRNAs. These miRNAs demonstrated a protective effect by enhancing survival, reducing apoptosis, and decreasing ROS accumulation in an acute kidney injury model (Toghiani et al., 2024). Similarly, in a study by Tscherrig et al. (Tscherrig et al., 2024), Wharton’s jelly mesenchymal stromal cell-derived small extracellular vesicles (WJ-MSC-sEVs) microRNAs (miRNAs) were shown to have a protective effect in an animal model of white matter injury when administered intranasally. However, in this study, the cells were not cultured under hypoxic conditions, despite the animal model being based on this condition. This again highlights the need for standardizing culture conditions if the therapeutic use of MSCs and their derivatives is to be optimized.

In relation to clinical findings, it is important to highlight that, despite promising preclinical results, clinical data on the efficacy of hypoxia-preconditioned MSCs remains limited. A search on clinicaltrials.org identified one study using hypoxic adipose tissue MSCs for the treatment of a posterior cruciate ligament injury, as well as two clinical trials employing conditioned media from hypoxic cultured MSCs to treat knee osteoarthritis and severe COVID-19; however, no results were available for any of these studies. This scarcity of data underscores that the full translation of preclinical findings into clinical practice still requires extensive research and validation. Nevertheless, a study on Parkinson’s disease (PD) in China presents promising results for clinical translation (Zhuo et al., 2024). This study demonstrated that hypoxia-preconditioned olfactory mucosa mesenchymal stem cells (hOM-MSC) improved functional recovery and reduced neuronal oxidative stress in a PD mouse model. Notably, in the translation clinical trial involving five PD patients, hOM-MSC transplantation resulted in a reduction in medication and improvement in motor function, as assessed by clinical scales and biomarkers. This case serves to illustrate the translational potential of the hypoxia preconditioning strategy and the modulation of key pathways. Despite these hopeful results in a clinical context, the need to conduct more clinical studies is emphasized to comprehensively evaluate the safety and efficacy of hypoxia-preconditioned MSCs in the field of regenerative medicine for various indications.

In conclusion, culturing MSCs under hypoxic conditions has emerged as a promising strategy to enhance their therapeutic potential by improving their proliferation, differentiation, immunomodulatory properties, and ability to migrate to injury sites. The molecular mechanisms underlying these effects, particularly the activation of HIF-1α and the upregulation of key growth factors and cytokines, provide valuable insights into how hypoxia can optimize MSC-based therapies. In parallel, exosomes derived from hypoxia-preconditioned MSCs offer a similarly promising strategy for enhancing regenerative therapies. The ability of exosomes to deliver bioactive molecules that regulate inflammation, promote tissue repair, and modulate cellular processes makes them an attractive alternative to traditional cell therapies. The synergistic effects of combining MSCs with hypoxia-derived exosomes further enhance the therapeutic potential of this approach, providing a powerful tool for treating a wide range of tissue injuries and degenerative diseases. As research in this field progresses, understanding the molecular mechanisms behind exosome production and cargo composition will be crucial for optimizing the use of these vesicles in clinical settings.

## Data Availability

The original contributions presented in the study are included in the article/supplementary material, further inquiries can be directed to the corresponding author.

## References

[B1] Armstrong J. P. K. Timothy J. K. Roques A. C. Stephen Patrick P. Mooney C. M. Kuan W.-Li (2020). A blueprint for translational regenerative medicine. Sci. Transl. Med. 12 (572), eaaz2253. 10.1126/scitranslmed.aaz2253 33268507 PMC7610850

[B2] Caplan A. I. (2010). Mesenchymal stem cells: the past, the present, the future. Cartilage 1 (1), 6–9. 10.1177/1947603509354992 26069532 PMC4440607

[B3] Dai T. Hu Y. Zheng H. (2017). “Hypoxia increases expression of CXCR4 leading to enhanced migration of EPCs.” 28485797

[B4] Fears R. Akutsu H. Alentajan-Aleta L. T. Caicedo A. Čolić M. (2021). Inclusivity and diversity: integrating international perspectives on stem cell challenges and potential. Stem Cell Rep. 16 (8), 1847–1852. 10.1016/j.stemcr.2021.07.003 PMC8365097 34329597

[B5] Franco J. P. Jaraba-Alvarez W. Blanquiceth Y. Bedoya A. Ortiz S. Uscanga A. (2025). Hypoxic culture vs. Normoxia standard culture in umbilical cord mesenchymal stem cells: enhanced exosomal yield and unique regenerative signature. Cytotherapy 27, S86. 10.1016/j.jcyt.2025.03.164

[B6] Galipeau J. Krampera M. Leblanc K. Nolta J. A. Phinney D. G. Shi Y. (2021). Mesenchymal stromal cell variables influencing clinical potency: the impact of viability, fitness, route of administration and host predisposition. Cytotherapy 23 (5), 368–372. 10.1016/j.jcyt.2020.11.007 33714704 PMC11708105

[B7] Glancy B. Kim Y. Katti P. Bradley Willingham T. (2020). “The functional impact of mitochondrial structure across subcellular scales,” 11. Front. Physiology, 541040. 10.3389/fphys.2020.541040 PMC7686514 33262702

[B8] Hu Z. Wang D. Gong J. Li Y. Ma Z. Luo T. (2023). MSCs deliver hypoxia-treated mitochondria reprogramming acinar metabolism to alleviate severe acute pancreatitis injury. Adv. Sci. 10 (25), e2207691. 10.1002/advs.202207691 PMC10477874 37409821

[B9] Keith B. Simon M. C. (2022). Hypoxia inducible factors. Stem Cells Cancer. 10.1016/j.cell.2007.04.019 PMC3150586 17482542

[B10] Khasawneh R. R. Abu-El-Rub E. (2022). Hypoxia disturbs the migration and adhesion characteristics of mesenchymal stem cells. Cell. Mol. Biol. 68 (11), 28–32. 10.14715/CMB/2022.68.11.5 37114312

[B11] Kwon Se Y. Chun So Y. Ha Y. S. Kim D. H. Kim J. Song P. H. (2017). Hypoxia enhances cell properties of human mesenchymal stem cells. Tissue Eng. Regen. Med. 14 (5), 595–604. 10.1007/s13770-017-0068-8 30603513 PMC6171625

[B12] Le B. Amin C. Morales D. Fierro F. A. (2024). “First clinical experiences using preconditioning approaches to improve MSC-based therapies,” 10. Curr. Stem Cell Rep., 1–7. 10.1007/s40778-023-00232-5

[B13] Liu Y. Tsai A.-C. Yuan X. Yan Li Ma T. (2017). “Hypoxia regulation of stem cell,” in Biology and engineering of stem cell niches (Elsevier), 273–291. 10.1016/B978-0-12-802734-9.00018-4

[B14] Lotfy A. AboQuella N. M. Wang H. (2023). Mesenchymal stromal/stem cell (MSC)-Derived exosomes in clinical trials. Stem Cell Res. and Ther. 14 (1), 66. 10.1186/s13287-023-03287-7 37024925 PMC10079493

[B15] Mas-Bargues C. Sanz-Ros J. Román-Domínguez A. Inglés M. Gimeno-Mallench L. El Alami M. (2019). Relevance of oxygen concentration in stem cell culture for regenerative medicine, Int. J. Mol. Sci. 20. 10.3390/ijms20051195 PMC6429522 30857245

[B16] Mohammadalipour A. Dumbali S. P. Wenzel P. L. (2020). “Mitochondrial transfer and regulators of mesenchymal stromal cell function and therapeutic efficacy,” 8. Front. Cell Dev. Biol. 10.3389/fcell.2020.603292 PMC7750467 33365311

[B17] Moll G. Ankrum J. A. Olson S. D. Nolta J. A. (2022). Improved MSC minimal criteria to maximize patient safety: a call to embrace tissue factor and hemocompatibility assessment of MSC products. Stem Cells Transl. Med. 11 (1), 2–13. 10.1093/stcltm/szab005 35641163 PMC8895495

[B18] Mu J. Li L. Wu J. Huang T. Zhang Yu Cao J. (2022). Hypoxia-stimulated mesenchymal stem cell-derived exosomes loaded by adhesive hydrogel for effective angiogenic treatment of spinal cord injury. Biomaterials Sci. 10 (7), 1803–1811. 10.1039/D1BM01722E 35234220

[B19] Mukkala A. N. Jerkic M. Khan Z. Szaszi K. Kapus A. Rotstein O. (2023). “Therapeutic effects of mesenchymal stromal cells require mitochondrial transfer and quality control,” in International journal of molecular sciences (Basel, Switzerland: Multidisciplinary Digital Publishing Institute). (MDPI). 10.3390/ijms242115788 PMC10647450 37958771

[B20] Pulido-Escribano V. Torrecillas-Baena B. Camacho-Cardenosa M. Gabriel D. Gálvez-Moreno M. Á. Casado-Díaz A. (2022). Role of hypoxia preconditioning in therapeutic potential of mesenchymal stem-cell-derived extracellular vesicles. World J. Stem Cells 14 (7), 453–472. 10.4252/wjsc.v14.i7.453 36157530 PMC9350626

[B21] Raposo G. Stoorvogel W. (2013). Extracellular vesicles: exosomes, microvesicles, and friends. J. Cell Biol. 200, 373–383. 10.1083/jcb.201211138 23420871 PMC3575529

[B22] Rodriguez A. M. Nakhle J. Griessinger E. Vignais M. L. (2018). “Intercellular mitochondria trafficking highlighting the dual role of mesenchymal stem cells as both sensors and rescuers of tissue injury,” in Cell cycle. London, United Kingdom: Taylor and Francis Inc. 10.1080/15384101.2018.1445906 PMC5969546 29582715

[B23] Samsonraj R. M. Raghunath M. Nurcombe V. Hui J. H. van Wijnen A. J. Cool S. M. (2017). Concise review: multifaceted characterization of human mesenchymal stem cells for use in regenerative medicine. Stem Cells Transl. Med. 6 (12), 2173–2185. 10.1002/sctm.17-0129 29076267 PMC5702523

[B24] Sánchez-Giraldo V. Jaraba W. Rivillas Y. Valencia-Rodriguez Y. Gómez-Cuesta M. Betancur-Rojas D. (2023). Mesenchymal stem/stromal cells: WHARTON’S jelly mesenchymal stem cells (WJ-MSCS) showed NEURON-LIKE differentiation when cultured in cerebrospinal fluid. Cytotherapy 25 (6), S62. 10.1016/S1465-3249(23)00235-9

[B25] Song H. Li B. Guo R. He S. Peng Z. Qu J. (2022). Hypoxic preconditioned aged BMSCs accelerates MI injury repair by modulating inflammation, oxidative stress and apoptosis. Biochem. Biophysical Res. Commun. 627 (October), 45–51. 10.1016/j.bbrc.2022.08.039 36007334

[B26] Teixeira F. G. Panchalingam K. M. Anjo S. I. Manadas B. Pereira R. Sousa N. (2015). Do hypoxia/normoxia culturing conditions change the neuroregulatory profile of Wharton jelly mesenchymal stem cell secretome? Stem Cell Res. Ther. 6 (1), 133. 10.1186/s13287-015-0124-z 26204925 PMC4533943

[B27] Terzic A. Pfenning M. A. Gores G. J. Michel Harper C. (2015). Regenerative medicine build-out. Stem Cells Transl. Med. 4 (12), 1373–1379. 10.5966/sctm.2015-0275 26537392 PMC4675513

[B28] Thomas M. A. Fahey M. J. Pugliese B. R. Irwin R. M. Antonyak M. A. Delco M. L. (2022). Human mesenchymal stromal cells release functional mitochondria in extracellular vesicles. Front. Bioeng. Biotechnol. 10 (August), 870193. 10.3389/fbioe.2022.870193 36082164 PMC9446449

[B29] Toghiani R. Zavareh V. A. Najafi H. Mirian M. Azarpira N. Abolmaali S. S. (2024). Hypoxia-preconditioned WJ-MSC spheroid-derived exosomes delivering MiR-210 for renal cell restoration in hypoxia-reoxygenation injury. Stem Cell Res. Ther. 15 (1), 240. 10.1186/s13287-024-03845-7 39080774 PMC11289969

[B30] Tscherrig V. Steinfort M. Haesler V. Surbek D. Schoeberlein A. Joerger-Messerli M. S. (2024). All but small: MiRNAs from Wharton’s jelly-mesenchymal stromal cell small extracellular vesicles rescue premature white matter injury after intranasal administration. Cells 13 (6), 543. 10.3390/cells13060543 38534387 PMC10969443

[B31] Wang Q. Li Y. Yuan H. Peng L. Dai Z. Sun Ye (2024). Hypoxia preconditioning of human amniotic mesenchymal stem cells enhances proliferation and migration and promotes their homing via the HGF/C-met signaling Axis to augment the repair of acute liver failure. Tissue Cell 87 (April), 102326. 10.1016/j.tice.2024.102326 38442547

[B32] Williams T. Salmanian G. Burns M. Maldonado V. Smith E. Porter R. M. (2023). “Versatility of mesenchymal stem cell-derived extracellular vesicles in tissue repair and regenerative applications,”, 207. Biochimie, 33–48. 10.1016/j.biochi.2022.11.011 36427681

[B33] Yang H.-ya Fierro F. Yoon D. J. Gallegos A. Osborn S. L. Nguyen A. V. (2022). Combination product of dermal matrix, preconditioned human mesenchymal stem cells and timolol promotes wound healing in the porcine wound model. J. Biomed. Mater. Res. Part B Appl. Biomaterials 110 (7), 1615–1623. 10.1002/jbm.b.35022 35099112

[B34] Yuan F. Liu J. Zhong L. Liu P. Li T. Yang K. (2025). Enhanced therapeutic effects of hypoxia-preconditioned mesenchymal stromal cell-derived extracellular vesicles in renal ischemic injury. Stem Cell Res. and Ther. 16 (1), 39. 10.1186/s13287-025-04166-z 39901252 PMC11792194

[B35] Yusoff F. M. Nakashima A. Ichiro Kawano Ki Kajikawa M. Kishimoto S. Maruhashi T. (2022). Implantation of hypoxia-induced mesenchymal stem cell advances therapeutic angiogenesis. Stem Cells Int. 2022, 6795274. 10.1155/2022/6795274 35355589 PMC8958070

[B36] Zhuo Yi Wen S.Li Wen Lu Li X. Ge Li Te Huang Y. (2024). TGF-Β1 mediates hypoxia-preconditioned olfactory mucosa mesenchymal stem cells improved neural functional recovery in Parkinson’s disease models and patients. Mil. Med. Res. 11 (1), 48. 10.1186/s40779-024-00550-7 39034405 PMC11265117

